# Opening science to society: how to progress societal engagement into (open) science policies

**DOI:** 10.1098/rsos.231309

**Published:** 2024-05-29

**Authors:** U. Wehn, R. Ajates, C. Mandeville, L. Somerwill, G. Kragh, M. Haklay

**Affiliations:** ^1^ IHE Delft Institute for Water Education, Westvest 7 Delft 2601DA, The Netherlands; ^2^ Raquel: Universidad Nacional de Educación a Distancia, Calle Obispo Trejo, n°2, Madrid 28040, Spain; ^3^ University of Dundee, Dundee, UK; ^4^ Centre for Biodiversity Dynamics, Department of Natural History, Norwegian University of Science and Technology, Høgskoleringen 1, Trondheim 7034, Norway; ^5^ Centre for Science Studies, Aarhus University, Ny Munkegade 118, Aarhus C 8000, Denmark; ^6^ Department of Geography, University College London, Gower Street, London WC1E 6BT, UK

**Keywords:** citizen science, open science, open data, open access, policy recommendations, societal engagement

## Abstract

A broad understanding of the aims and objectives of the international open science movement was recently adopted with the 2021 UNESCO Recommendation on Open Science, expanding the focus of open science to include scientific knowledge, infrastructures, knowledge systems and the open engagement of societal actors. In response, recent discussions on science policy practice are shifting to the implementation of open science via national policies. While policy instruments to support some aspects of open science are well-studied, guidance on the emerging ‘social’ aspects of open science has lagged, prompting UNESCO to generate guidance. In this paper, several authors of the UNESCO Open Science Toolkit guidance document on ‘Engaging societal actors in Open Science’ synthesize the scholarly underpinnings behind its recommendations. This work draws upon a targeted search from academic, policy, and grey literature in the fields of open science and community engagement, with a special focus on citizen science, to derive guidance on how to overcome barriers to the uptake of societal engagement approaches. The results present building blocks of what an enabling environment for the open engagement of societal actors could look like, identifying key considerations and reflecting on opportunities and challenges for progressing and evaluating sound open engagement of societal actors into regional & national (open) science policies.

## 1. Introduction

While policy instruments to support some aspects of open science are available—including open access and open data—guidance on the emerging ‘social’ aspects of open science has lagged. The 2021 UNESCO Recommendation on Open Science's identification of ‘Open engagement of societal actors' as a pillar of open science has prompted increased focus on social aspects of open science, resulting in the development of a specific guide in the UNESCO Open Science Toolkit, summarizing regional and national policy recommendations to support the open engagement of societal actors. Here, the authors of this guide on engagement of societal actors in open science report, expand and reflect upon the scholarly underpinnings behind the guidance document's recommendations. Our general aim is to support the application of the guide by different stakeholder groups. The guide was addressed to policy makers and policy professionals who are less familiar with the field, to benefit from understanding the place of citizen science within the wider field of Open Science. Through this paper, researchers and practitioners can access the additional details that clarify the underlying work that led to the guide, with the view of informing their open science practice, and for the OS guidance document to be built upon, critically evaluated, and improved over time. In 2019, the 193 UNESCO Member States tasked the organization with the development of an international standard-setting instrument on Open Science in the form of a UNESCO Recommendation.

The UNESCO Recommendation on Open Science was adopted by the General Conference of UNESCO at its 41st session, in November 2021,

‘Noting the transformative potential of open science for reducing the existing inequalities in STI [i.e. science, technology & innovation; added] and accelerating progress towards the implementation of the 2030 Agenda and the achievement of the Sustainable Development Goals (SDGs) and beyond’.

‘Considering that more open, transparent, collaborative and inclusive scientific practices, coupled with more accessible and verifiable scientific knowledge subject to scrutiny and critique, is a more efficient enterprise that improves the quality, reproducibility and impact of science, and thereby the reliability of the evidence needed for robust decision-making and policy and increased trust in science’ [p.2].

‘Open Science’ is an umbrella concept, which includes a range of ‘open’ labels of research practices, such as Open Source Software, Open Access to publications, Open Educational Resources, Open Data, as well as opening the process of scientific knowledge production to other actors, or ‘citizen science’ in its dual value of science produced by citizens—through the collection of data and involvement in other steps of the scientific method—and science made for citizens, or scientific communication.

Open science emerged in the late 2000s, as a solution to address recognized issues in scientific practices (such as the difficulties in accessing scientific papers) and by taking advantage of emerging web technologies (see for example [1,2]). Its original formation highlighted open scientific data as a core principle, joined by open-access publication and open-source scientific software, as well as openness to the public in terms of their engagement in scientific practice. In practice, most attention was paid to the aspects that are at the core of the concerns of scientists such as Open Access and Open Data. Since then, the concept evolved, with an adoption by the European Commission as a central part of research policy [3], with eight areas of activity: rewards and incentives, indicators and next-generation metrics, future of scholarship, European Open Science Cloud (EOSC), FAIR data, research integrity, skills and education, and citizen science [4]. Eventually, global awareness, attention and some form of consensus converged and materialized in the form of the UNESCO Recommendation on Open Science. This Recommendation was motivated by the need to change ‘science as usual’ in order to achieve the Sustainable Development Goals (SDGs) and was adopted by all Member States in November 2021.

UNESCO Recommendations are legal instruments emanating from the organization's supreme governing body, and hence, recommendations are set to shape and influence national laws and practices [5]. With the UNESCO Recommendation now in place, recent discussions on science policy practice are shifting to the implementation of open science via national and regional policy. Several countries have already undertaken concrete steps to shaping their existing science, research and innovation policies. As De Filippo & Sastrón-Toledo ([6], p.2) have reported, in Europe, Finland has its Open Science and Research Initiative [7,8]; the Netherlands, the National Plan Open Science [9]; Portugal adopted an open scientific policy [10], France launched the French Plan for Open Science in 2018 [11], Greece kicked off the National Open Science Plan for Greece in 2020 [12]. Recently, the National Plan for Open Science was developed in Italy [13], Austria approved its policy on Open Science and the European Open Science Cloud in 2022 [14] and Spain introduced the National Strategy for Open Science 2023–2027 [15].

The efforts in these countries are characterized by an evolution of openness, with earlier open science policy efforts focusing on open data, open access and open infrastructures, while more recent policy efforts include citizen science and other participatory approaches. In some countries, open data policies are a prior policy development that seems to evolve into open and participatory policies [6]. For example, in the case of Spain, its 2017–2020 State Plan on Scientific and Technical Research and Innovation made it mandatory for results and research data obtained under public funding to be open [16]. In 2022, the country's new Act 17/2022 of 5 September on science goes a step further: ‘to facilitate free access and management of data produced through research (open data), in accordance with international FAIR principles (Findability, Accessibility, Interoperability and Reusability), to develop open infrastructure and platforms, to foster the publication of scientific results in open access and civil society's open participation in scientific processes' ([17], cited in [6]). At European Level, the year-long Mutual Learning Exercise (MLE) on citizen science Initiatives by 11 countries that ended in January 2023 has been a key forum for policy makers in the area of R&I policy and funding to learn about citizen science and how to embed this into their policies. During the year, policy makers exchanged information about their policy development, which was enhanced through site visits in Austria, Germany, Slovenia, Belgium and Hungary. The MLE provided the space for online and face-to-face long discussions guided by experts and information sharing. At a global level, from the interactions in the UNESCO Open Science Working Groups, it is becoming increasingly clear that the ‘social’ pillars of the Recommendation are still lacking attention, i.e. the conditions for creating an enabling environment for citizen science and other forms of open engagement.

From the beginnings of the development of the open science concept, the opening up of the scientific process to actors who are not professional scientists was recognized. For example, Nielsen [1] recognized that citizen science, mostly in the form of crowdsourcing activities such as Galaxy Zoo, a project in which volunteers help astrophysicists to classify images from telescopes, is changing the relationships between science and society. Boulton *et al*. [2] recognized that ‘The growth of the citizen science movement could turn out to be a major shift in the social dynamics of science, in blurring the professional/amateur divide and changing the nature of the public engagement with science. Free or affordable access to scientific journals and data would provide important encouragement to the movement’ (p. 40). Yet, there is a need to notice that citizen science has a different place within the family of practices and principles that are part of open science. Many of the open science principles are about the way scientists do science, such as opening datasets, using replicable open-source software, or publishing their papers in open-access repositories. All these actions do not change the fundamental process of ‘doing science’ as a process that happens among specialists—a dataset can be shared in a highly specialized format, and the open-access publication can be written in a niche jargon. These principles also fit into what Mačiulienė [18] terms the quality-control nature of open science. It is therefore unsurprising that this set of changes is easier to implement within the current science system. Citizen science, on the other hand, is about opening the relationships between science and society [18] and as a result, requires a change in the role of scientists and the way they do science. As Golumbic *et al*. [19] and Riesch and Potter [20] evidence, the acceptance of citizen science by scientists is challenging as it requires a change of practices and epistemic approaches. This challenge is also noticeable in policy documents and the academic literature, with a much bigger emphasis on open access, open data, and open source than on societal engagement, a focus that is also reflected in funding allocation.

Furthermore, we need to be aware of the critiques of open science and citizen science and not present them as panaceas. As Ross-Hellauer [21] pointed out, there are significant costs associated with maintaining high-quality open science practices and these might increase inequality and the ability to do and publish science. A much wider critique is provided by Mirowski [22] where he highlights the potential for exploitation of people's effort within a scientific process that is ultimately aimed at generating profit-making products or services, highlighting the need to consider who benefits from open science approaches. Mirowski [23] has also levelled a specific critique on citizen science and the degradation of expertise that it can lead to, as well as a way to justify cutting funding and resources from scientists. Issues around the diversity of citizen scientists have also been identified, as many citizens lack the time, skills and/or confidence to take part in citizen science projects, raising calls for more inclusivity [24].

The purpose of this paper is to detail and reflect upon the scholarly underpinnings of the guidance document of the UNESCO Open Science Toolkit on ‘Engaging societal actors in Open Science’ in light of this nuanced understanding of open science and citizen science. First, we provide further context on the process of synthesizing diverse examples from the field of citizen science that were used to derive building blocks for progressing the open engagement of societal actors into (open) science policies. Second, we discuss the key elements identified as building blocks of an enabling policy environment for open engagement of societal actors. Finally, we offer new reflections on the potential cascading impacts of opening science to society within the science policy community, considering our synthesis in light of current literature to discuss future opportunities and challenges.

## 2. Background and method

Open engagement of societal actors constitutes a landscape of approaches and practices that involve, or are initiated by, stakeholders across society. One of the most prominent forms is citizen science. While this term is often understood to primarily involve the crowdsourcing of data collection or data processing by volunteers, in practice it entails many forms of collaborating with members of the public in knowledge co-creation and application. A recent study capturing the views of 333 citizen science practitioners highlighted the pluralities of citizen science and that context-specific definitions are needed [25]. Common characteristics of different interpretations [26,27] are that citizen science:

— Involves participants in one or more steps of the scientific research process;
— Is practised across all areas of research and knowledge production, from environmental to social or health conditions monitoring;
— Is applicable across all scientific and scholarly disciplines, alongside a variety of disciplinary traditions and research methods;
— Follows protocols and principles of the discipline within which the research is framed; and,
— Varies in terms of the roles, responsibilities, and leadership opportunities for scientists, their fellow citizens, and other stakeholders.

The diverse societal engagement practices display a range of initiators as well—from science-driven ones, to community-driven and those initiated by authorities and monitoring agencies—along with a range of purposes (such as scientific investigation, conservation, local action and education) (see box 1). All societal engagement approaches embody different notions of expert knowledge and are affected by structures that influence science dynamics at work, including which questions are asked, which methods are used, how data are shared and who can access and analyse any data collected. The diversity of societal engagement approaches is indicative of the changing role of science in society and the shifting interactions between science, society and policy, illustrating that universities no longer have the monopoly over knowledge production. Societal engagement initiatives are embedded in and responsive to a number of dynamics and aspects: thematic, geographical & temporal dimensions; socio-political context; scientific discipline(s); technological dimension; and financial resources [39].

Box 1 **. Different forms of open engagement of societal actors.**

**Citizen science**: Participation, in whole or part, in the scientific life cycle by those who do not hold credentials typically associated with that field of science [25,28].

**Community-based participatory research**: Collaboration with communities most affected by an issue, enlisting them to conduct research, and devising solutions together, often in a health, public health, or environmental context [29].

**Community science**: Science initiated by communities underserved by scientific institutions, and elevating local expertise and issues above academic interests [30].

**Community-based monitoring**: Routine observations of environmental or social phenomena, or both, that are led and undertaken by community members and civil society associations, and can involve external collaboration and support of visiting researchers and government agencies [31].

**Citizen observatories**: Community-based environmental monitoring and information systems, that invite individuals to share observations, typically via mobile phone or the web. Emphasises two-way flow of information and citizens conducting environmental monitoring [32].

**Crowdsourcing**: Distributing a discrete set of tasks—such as data collection or processing—among participants, often used to lower costs or increase the speed of research for those running a project [33].

**Listening at scale/ implicit sensing/ social media listening**: Employing data science methodology—including machine learning and natural language processing—to surface consensus from differing opinions among a large population [34].

**Participatory action research**: Involving an affected community throughout research with a specific problem to solve, often used in the social sciences and sometimes includes taking political action [35].

**Science shops/Citizen science labs**: Mechanism (typically of universities) for initiating challenge-driven research defined by citizens and communities and undertaken with and by scientists [36].

**LivingLabs**: Innovation experiments with stakeholders of the quadruple helix (academic, private sector, public sector, civil society) in their real-life setting [37]. The extent to which LivingLabs serve to open up the scientific process varies greatly, however.

*Source*: based on Wehn *et al*. [38].

Attention to the governance of societal engagement processes is important to ensure more egalitarian practice and knowledge co-production. Lack of awareness, acceptability and sustainability are well-known barriers to the broader uptake of societal engagement approaches such as citizen science and citizen observatories [32,40]. An enabling environment for the open engagement of societal actors and dialogues with other knowledge systems promotes awareness of their potential, validation of their contributions to science, acknowledgement of and willingness to address policy and societal challenges, and sustainability for their activities. Open science policies are key for establishing this environment and for leveraging the potential of societal engagement in science to address key societal challenges, especially those encompassed in the SDGs. Existing research on enabling environments for citizen observatories (CO) based on citizen science has demonstrated that an enabling environment consists of ‘the sum of conditions that enable a CO to start, function and sustain its activities to deliver value and impact across multiple stakeholders' (p.21), featuring active and engaged networks of stakeholders; skills, capacity building and training; suitable and reliable technology, infrastructures and data policies; and flexible legal and policy frameworks to ensure impact and sustainability [32]. Our discussion builds on a collaborative effort of a global network of citizen science practitioners carried out in autumn 2022 that captured existing insights and recommendations on what policy makers can do to shape their (open) science policies in order to ensure the open engagement of societal actors in science [41]. These insights were collated from relevant academic literature that included concrete policy recommendations, policy briefs and other policy-oriented documents, as well as project deliverables, conference papers, and funding agency guidelines. Targeted searches in the academic and grey literature were carried out for policy recommendations on how to embed citizen science and other forms of open engagement of societal actors in open science policy, complemented by the authors’ own knowledge of existing materials, accompanied by a broad call for participation within relevant professional communities, resulting in 52 documents for the detailed analysis. Following on from an initial search for inputs, we identified thematic and geographical gaps and followed with a snowballing approach to complement the dataset. This approach was effective as the authors are experts working on open, participatory and citizen science topics. The resulting documents were analysed with a view to extracting recommendations in the seven action areas that are mentioned in the UNESCO Recommendation on Open Science, namely, a common understanding of open science; an enabling policy environment for open science; investments in open science infrastructure and services; investments in human resources, training, education, digital literacy and capacity building; fostering a culture of open science and aligning incentives for open science; promoting innovative approaches for open science at different stages of the scientific process; promoting international multi-stakeholder cooperation for open science and reducing digital, technological and knowledge gaps [42].

This resulted in a structured data set which was cleaned to identify duplicates resulting in a data set of 33 documents; the original documents were checked in cases when clarifications of the extracted entries were needed. A thematic analysis was undertaken to synthesize these inputs into specific policy recommendations in four specific areas related to opening science to society: i) open understanding; ii) capacity building; iii) infrastructure and services; and iv) funding from which policy recommendations for governments were derived.

## 3. Progressing citizen science via (open) science policy

Given limited existing guidance for progressing open engagement of societal actors via policy, this section presents and contextualizes specific policy recommendations for embedding citizen science and other forms of open engagement of societal actors in (open) science policy (table 1). Drawing on the knowledge and insights of the citizen science community, the presented policy instruments related to the creation of an open understanding of the open engagement of societal actors, capacity building at different levels, infrastructure and services for open engagement of societal actors, and funding mechanisms.

**Table 1.** Summary of recommendation for opening science to society via open science policy. *Source:* Wehn *et al*. [43].

**open understanding of opening science to society**	**empower and engage societal actors through open engagement**	— go beyond facilitating public participation in scientific processes that are led top down — foster transparent communication & long-term relationships with community partners — ensure free and open access to educational content, enhance science and data literacy
	**ensure diversity, equity, inclusion, & justice in opening science to society**	— ensure engagement of and partnerships with marginalised communities — support non-traditional venues for scientific activities and accessible communication — ensure benefits of societal engagement reach all involved stakeholders
**capacity building on opening science to society**	**national & policy-maker levels**	— create enabling environment that cut across governance levels — leverage existing resources — foster multi-level and multi-stakeholder policy connections
	**institutional & individual level**	— foster capacity building and academic recognition within Higher Education Institutions — foster societal engagement through (high) schools and life-long learning programmes — support informal training initiatives
	**knowledge exchange opportunities**	— prioritise impact at scales from local to global — support development of infrastructures for practitioners of open societal engagement — ensure accessible resources
**infrastructure & services for opening science to society**	— develop online infrastructure for societal engagement — incorporate Open Data sharing — encourage reusability and interoperability by developing standards that require input from societal actors — support bottom-up development of infrastructure to allow societal actors to shape tools for engaging with science	
**funding for opening science to society**	**for what**	— foster open societal engagement and dialogues with other knowledge systems — mainstream societal engagement in all funding
	**for whom**	financial support for a wide range of actors
	**for how long**	funding models to focus on creating diverse, long term relationships & community building
	**by whom**	collaboration between public and private funding agencies
	**fit-for-purpose instruments**	specifically address quality criteria of good societal engagement & co-create fit-for-purpose funding & evaluation instruments

### 3.1. A more open understanding of engagement in science with societal actors

Open engagement with societal actors offers great potential for communities to take a leading role in scientific knowledge generation, interpretation, and implementation [44,45]. A supportive policy environment can facilitate a move beyond top-down leadership of societal engagement in science and towards empowerment of societal actors. For instance, policies can support long-term relationships between societal actors and relevant scientific and government institutions and can establish communication practices that centre transparency, openness and listening [45,46]. To take into account the multiple interrelated values and priorities of societal actors often requires that regular consultations with relevant communities, especially those that are typically under-reached, take place iteratively throughout the definition, launch and implementation of project work, and requires openness to reframing the goals or focus of the initiative to address community priorities [47]. This is especially critical when multiple relevant communities have priorities that vary or may even be at odds.

Striving to align scientific processes with community priorities by translating national- and global-level targets to local contexts can make science policies more effective [48,49]. Trusted partners and organizations can be engaged to serve as a bridge between national-level policies and local-scale implementations; potential partners include natural resource managers, libraries, museums, community centres, higher education institutions and tribal institutions [45,47,50–52]. To establish these diverse partnerships, funding strategies need to encompass a wide range of facilitating organizations and communication channels. Communication methods need to be broadly accessible, adhering to principles of universal design [53]. Reciprocal engagement with societal actors further necessitates open dialogue and engagement with other knowledge systems. Traditional Ecological Knowledge (TEK) systems should be valued in their own right [52,54,55] and Indigenous guidance should be followed regarding scientific knowledge exchange and data sovereignty [56].

Effective empowerment of societal actors is supported by continuous investment in science education and data literacy [49,57]. Policies can support free and open access to high-quality educational content and opportunities [42,58]. Such investments in public education can be implemented through public institutions such as schools and libraries and can also be explicitly built into citizen science and other forms of societal engagement [45,50]. Evidence from completed citizen science projects indicates that such investments in public data literacy can further facilitate the emergence of community-led participatory scientific activities [49,59].

Policies must be intentionally designed to seek to understand the needs, motivations, and effective means of engagement with communities that have been disenfranchised. To date, Black and Indigenous communities and other communities of colour have been largely marginalized, and are under-represented in large-scale science engagement and citizen science efforts [24,46]. Policies that support the engagement of marginalized communities should enact systems to monitor and ensure that participating communities will receive reciprocal benefits from participation [46]. Globally, open science can break down barriers that separate scientists from rich countries from those from poor countries, and this outcome must be actively fostered in open science policies [47]. Access barriers that may limit scientists from economically disadvantaged countries from fully participating in open science, including limited technological and computational resources, disproportionate opportunity costs and lack of career benefits, must be addressed [60,61]. Having inclusion or fairness as objectives should start paving the way to achieving them, as with other project objectives, receiving personnel, financial and monitoring resources. And in the cases in which they are not achieved, at least this will highlight shortcoming and, being formalized as objectives, will facilitate dedicating the project resources to identifying the reasons for not meeting them. The UNESCO Recommendation aims to make these objectives visible and legitimate.

### 3.2. Capacity building on opening science to society

A culture of societal engagement can be fostered in many ways by embedding the concept within internal and external training, outreach, and support at all policy levels. Awareness raising, education, training, knowledge exchange, and availability of resources are the foundations to build mutual trust between policymaking institutions and the public, which is an enabling condition for societal engagement [32]. Long-term support for capacity building will better sustain ongoing leadership, coordination, and legitimation for the ever-widening range of societal engagement methods available [62]. Key stakeholders may be encouraged to support and implement capacity building activities, with training in both formal and informal contexts adapted to a diverse range of stakeholders [63].

#### 3.2.1. Coordinating capacity building across scales

Open engagement of societal actors and dialogues with other knowledge systems will look different across various government institutions; therefore, national-level policy is a suitable lever to facilitate the explicit integration of open engagement into all relevant institutions that directly implement societal engagement. National policies can include measures to equip all relevant staff (such as local, regional, and national government officials and scientist-regulators) with sufficient capabilities to carry out effective open engagement and to encourage them to become proponents of societal engagement approaches, become familiar with citizen potential in science, and share success stories [50,57]. Local government officials, in particular, are the closest and generally the first contact that citizens have with the government; thus, staff with training in communication, training and support, and interacting with local organizations and citizens are a valuable asset [50,64]. Additionally, all relevant staff and institutions can be integrated into a cross-cutting community of practice, which can be supported by one or more dedicated positions that serve as a primary point of contact for open engagement [45].

National-level (open) science policy can produce cross-scale impacts by facilitating the sharing of resources and best practices between the local actors responsible for much of the practical work of open engagement. Furthermore, it can establish common frameworks, promote synthesis among institutions, and help facilitate cross-cutting opportunities [45,65], generating a policy environment that is responsive to emerging best practices.

#### 3.2.2. Capacity building at the formal education, institutional and individual level

The adoption and institutionalization of open science and specifically the engagement of societal actors and dialogues with other knowledge systems requires strategic support in higher education institutions and other research-performing organizations, as well as schools, colleges, and life-long learning organizations [52,66]. A mechanism to enable this process is the inclusion of open science topics such as community-based participatory research, citizen science, and open access, in general research education and training curricula, particularly in postgraduate courses [29,47].

Ministries of Education are in a good position to initiate this integration of citizen science into higher education curricula and teacher training as a format for experience and research-based learning. This will require the development of updated teaching and learning materials [57,63]. Additionally, a culture of recognition, education and training can help overcome scepticism [57], alongside the adoption of new performance metrics and criteria that reward open engagement [62]. At institutional levels, structures and strategies can be adapted to provide services and guidance regarding societal engagement, further developing a culture of engagement [57]. A range of other recognition instruments can also be developed in collaborations between ministries, authorities, citizens, and research institutions (e.g. citizen science awards and revised university rankings) [57]. Citizen science topics can be incorporated into any level of education, and many examples exist of how it can be embedded in educational programmes at early stages with children as a way of increasing scientific literacy and incentivizing future citizen scientists [67–69].

As well as formal education institutions, evidence suggests that informal training providers and initiatives, such as national associations, museums, Citizen Observatories, and individual citizen science projects engaging with societal actors—especially trusted actors in local communities—often run important and successful capacity-building initiatives [50]. Importantly, open education can support more open access and utilization of open data. Capacity building among citizens and interested communities is essential so that they can enjoy the benefits of the available data, e.g. learning to interpret water quality data of their local river, air pollution or soil data [49,50,70]. Otherwise, only groups and companies with the right mix of skills can benefit from and capitalize on open data. Open engagement initiatives that receive strategic and financial support to facilitate training of diverse stakeholders, including local organizations, marginalized groups, and local community members, can help the development of the required capacity building.

All levels of informal and formal education and training institutions will benefit from cross-institutional agreements with existing associations, groups, civil society organizations, and schools, as they enable greater interaction with education, societal actors and the general public, e.g. through Science Shops or collaboration with teachers and schools.

#### 3.2.3. Knowledge exchange opportunities

Given the parallel development and evolution of (open) science policies at different levels (from local to global), careful alignment is required to ensure that policies supporting open societal engagement at a national level are coherent with international standards for open science. This will allow for leveraging of resources, sharing of best practices, and synthesis of policy outcomes across national boundaries. An example of how such alignment can be fostered are the interactions of various national policy makers from Austria, Belgium, France, Germany, Hungary, Italy, Norway, Portugal, Romania, Slovenia and Sweden in the recent MLE on Citizen Science [71]. At the same time, it is crucial that such international alignment does not inadvertently introduce barriers to local implementation, a risk which can be mitigated by involving local societal stakeholders early and frequently in policy development. Inherently, such engagement would need to be inclusive and accessible to all relevant stakeholders, providing accessibility in non-technical language and the offer of technical assistance or specific training where relevant.

Sharing of experiences between stakeholders and projects and providing opportunities for synergies and collaborations can be supported by the development of dedicated platforms, infrastructures, and fora for practitioners of open societal engagement [45] and interactions with existing networks (e.g. C40 cities). This can help identify and propagate the benefits and impact of engaging societal actors [45]. The creation of networks and relationships with and between diverse groups requires material support. One example of such support at a national level is the Sparkling Science program (www.sparklingscience.at) of the Austrian Federal Ministry of Education, Science and Research, which funds and sustains infrastructure for citizen science initiatives within Austria. Similarly, communities of practice at national level, supported by dedicated points of contact, can promote engagement between actors, while national-level monitoring of societal engagement efforts can serve to distill lessons and best practices to help actors adapt and improve open engagement over time [45]. Partnerships for open societal engagement, including between the Global North and the Global South, are useful vehicles for sharing best practice and strengthening the capacity to implement citizen science practices and can focus on concrete actions (e.g. to facilitate the implementation and advancement towards the SDGs). In general, the management of platforms, infrastructures, and fora for knowledge exchange should include ongoing assessment based on clearly defined evaluation metrics [57].

A wealth of resources, such as guidance, outreach materials, technology and data handling and processing recommendations, as well as the state of the art in collaborative approaches (such as participatory design methods) already exists. Dedicated repositories targeted at specific stakeholders can help ensure their access and use. Similarly, open access (OA) and open data are mechanisms that facilitate access to scientific materials and methodologies to a wide range of societal actors, hence support for OA publications and open data sharing are needed [72–75]. Policymakers can further consider the potential for partnership with the growing number of national and international nonprofit organizations and professional associations whose purpose is to build community and infrastructure around societal engagement in science (e.g. the European Citizen Science Association, the Association for Advancing Participatory Sciences, the Global Citizen Science Partnership, the Thriving Earth Exchange, the new Citizen Science Association Southern Africa).

### 3.3. Infrastructure and services for opening science to society

The development of online infrastructure (either at national or regional level, provided by government or in a more bottom-up manner) for societal engagement is required to enable exchange between and across initiatives, host interactive and step-by-step toolkits—such as the WeObserve Cookbook [32,76]—and can operate as a screening system for purpose-specific search for projects in line with quality standards. Platforms such as EU-Citizen.Science offer a template for how this can be done.

Open data sharing can also be incorporated into centralized infrastructures [66]. In order to allow for citizens' involvement in data analysis and visualization, accessible infrastructure is required to process data generated by societal engagement initiatives (especially environmental, land use, urban structure, socio-economic, Earth observation and other geodata) and transfer them to a common spatial data infrastructure or data catalogue [63]. This recommendation can be achieved at different levels. An international example is the Global Biodiversity Information Facility, demonstrating a current approach to international open data infrastructure in the context of biodiversity data [77]. A national example is offered by the ‘Italian Competence Centre on Open Science, FAIR, and EOSC’. The Competence Centre ICDI-CC is an initiative born within the Italian Computing and Data Infrastructure (ICDI), a forum created by representatives of major Italian Research Infrastructures and e-Infrastructures, with the aim of promoting synergies at the national level, and optimizing the Italian participation to European and global challenges in this field, including EOSC, the European Data Infrastructure (EDI) and HPC [78]. Substantial progress can also be achieved at the local level, e.g. Aarhus Municipality has developed a lot of experience running participatory science projects and championing open data, including inspirational cases, and outlining very practical guidelines for implementing CS projects [79].

At a project level, an example of data integration and participatory science was provided by the WeObserve projects, which further developed innovative Earth observation technologies and applications that enabled citizens to effectively participate in environmental stewardship and express the policy priorities of their community [32]. To achieve this at a global level, the Global South requires better Internet access and shared, community-governed and not-for-profit digital infrastructure for their citizens, as well as researchers and universities.

Reusability and interoperability can be encouraged by developing standards that require input from societal actors on the reuse, repair and further development of existing technologies [32]. Promoting open-source software and hardware, shared code bases, and sustainable hardware is central to including societal actors in science and supports initiatives through the availability of a richer set of features and functionalities that can be applied and adapted to other contexts. At the university level, administrative infrastructure and resources, such as the support of Data Stewards and Data Competence Centres, can provide key support for community-university research partnerships that empower people of all abilities to make and use accessible, open-source technologies.

Bottom-up development of infrastructure can allow citizens and societal actors to shape the tools that they will use when engaging with science. This can be done with the use of (digital) ‘living labs’, Digital Innovation Hub Networks and sandboxes to allow for collaboration and experimentation [80]. Additionally, the available infrastructures can then also be used to give access to small research groups, technology centres and companies.

### 3.4. Funding for opening science to society

Citizen science and other forms of open engagement of societal actors require careful consideration of how funding is made available, placing new demands and opportunities on funding instruments, including what activities and who should be funded, the length of time projects need to be funded for, where funding could or should come from, and how evaluation processes need to be shaped.

By their very nature, citizen science initiatives and projects are characterized by the involvement of citizens, communities and other stakeholders. Regardless of who takes the leading role (e.g. science-led, community-led), these projects incur relatively high start-up costs to identify and engage relevant stakeholders to be involved, to keep them motivated, and for their needs to be identified via co-design processes, as well as identifying and measuring baseline indicators that will help in conjunction with evaluation efforts at the end of the project to ascertain the impacts achieved [45,81,82]. Citizen science practitioners have highlighted the need for innovative funding instruments that welcome project proposals that provide flexibility for including scoping phases and the co-design of research agendas which can result in ‘yet to be defined’ outcomes based on such societal engagement; and for accepting changes to project execution [81].

In order to mainstream societal engagement in all research, it has been argued that the full range of research grant programs available needs to be adapted to reward participatory methods [45], encouraging various forms of societal engagement in science [59]. Strategic and financial support is necessary for research collaborations between communities and universities (as exemplified by Canada's many programmes on partnership research) [47,66], citizen science networks, capacity-building activities and initiatives [66], dedicated coordination and support actions across projects implementing open engagement of societal actors to foster peer-learning [32], as well as to changes in research organizations [66].

The open engagement of societal actors implies that a wide range of actors requires funding support, including small, local initiatives that can help engage local communities, grassroots and community-based organizations in science [59], with funding made available to community-based organizations, community-based partners (civil society organizations as well as citizens) as the direct recipient of grant awards [29]. Allowing community-based organizations, small businesses and other non-academic research organizations to serve as principal investigators for citizen and participatory science projects requires streamlining the procurement process to make it easier for them to submit proposals as well as providing guidelines, templates and tutorials for procurement of necessary services, tools and resources to support citizen science and crowdsourcing projects [52].

The longer term nature, ambitions and ethical responsibilities of many forms of open engagement of societal actors mean that operational and maintenance costs can extend beyond the end of defined project funding, especially when an engaged community of participants wishes to continue to monitor a local issue of importance to them [81]. This places demands on funding models to recognize the focus on creating relationships with diverse stakeholders and on community building [29], the longer time periods over which these forms of societal engagement are typically created and operate [81], allocating appropriate long-term funding to support sustainability [82].

Given that, in the short run, the costs of open engagement of societal actors are often higher than ‘traditional’ approaches, this places additional demands on research funding budgets. The co-ordination of alternative sources of funding with more traditional ones, with public-private co-financing and philanthropy collaborating to develop and implement co-sponsored grant initiatives that foster societal engagement in science, is reliant on a regulatory environment (laws of patronage, crowdfunding, venture capital with attractive taxation) that facilitates and stimulates private investment in research and innovation that embraces the open engagement of societal actors [80], provides innovative funding schemes and funding support functions [32], as well as guidance on how to obtain direct funding as well as other funding [59].

The review criteria for judging applications and final evaluations of projects practising the open engagement of societal actors need to recognize a wide range of success criteria, including but not limited to traditional measures of scientific quality [63]. To ensure that funding instruments and evaluation processes and criteria are fit for purpose, it has been argued that they could be best accomplished by co-creating them together with the stakeholder groups that they aim to serve, who have experience with the unique needs and considerations of societal engagement initiatives in those contexts [63]. The persons involved in the review process should be consistent with societal engagement principles themselves, making sure that the input of community participants in the review process is heard and incorporated into the final decision.

## 4. Opportunities and challenges ahead for opening science to society

Aside from changes in the content of open science policy itself, the multilevel and multi-actor changes required for open engagement of societal actors represent a transformation in the manner the scientific and policy communities ideate, shape and monitor (open) science policies. These changes will have cascading impacts, introducing new skill sets, new ways of approaching Knowledge Transfer (KT), and new knowledge systems and impact evaluation. These changes present cross-cutting opportunities and challenges, which we reflect upon in this section.

### 4.1. New skills and infrastructure to strengthen engagement of communities with science

Open engagement of societal actors calls for the development of new skills across many societal sectors, as discussed in the capacity building section above. These skills expand upon those highlighted in current discussions on open science, which currently centre on skills and capacity for open data. This finding directly links and is in line with the research from the EU Working Group on Skills for Open Science, which has identified four categories of open science skills and expertise: (1) skills for Open Access publishing (e.g. selecting a publishing venue and appropriate licensing; (2) data sharing and reuse skills, including standards for the formatting and curation of data and metadata; (3) disciplinary and research ethics skills that preserve research integrity and abide with the law (e.g. aligning openness with the data protection legislation); and (4) skills needed to engage the general public in research planning and activities (citizen science skills) [83]. The Group has highlighted how these skills can constitute an incentive for researchers interested in open science and can contribute to career visibility and progression. Some studies suggest that citizen science approaches even open demand for a new set of professionals who specialize in facilitating citizen participation in scientific processes [84].

In terms of infrastructure, evidence suggests that research infrastructure for social sciences research often lags behind that of natural sciences [80], and societal engagement projects such as citizen science experience the same fate. Funding for inclusive maker spaces, fab laboratories, citizen science training hubs, policy laboratories and other social infrastructure [85] can support the correct implementation of the UNESCO Recommendation.

In addition, societal engagement in science opens opportunities for dialogue between communities and other actors in the scientific process. Community science and community-based monitoring are considered to have the most explicit ‘bottom up’ approaches. However, through appropriate design, top-down projects might also generate bottom-up activities and impacts [49]. Societal engagement approaches present diverse opportunities for dialogues with other knowledge systems, e.g. by including and elevating local, traditional and Indigenous Knowledge. The extent to which this is achieved, and in what ways, is subject to evolving best practices and requires careful and sustained attention. Other forms of civic participation exist that may include practising open science, but might not be labelled by their initiators or others to be citizen science or participatory science—these should also be considered when describing societal engagement with science.

### 4.2. Risk for the valorization of science and participants' efforts

One of the key concerns about the effects of public participation in science is that funding for professional experts will be removed or reduced, with vested interests using calls for citizen participation as an excuse to shift responsibility for monitoring and data collection and analysis from the state onto civil society [23,86].

A comprehensive understanding of opening science to society should acknowledge that citizen participation is not a zero-sum game that will necessarily save research costs; in fact, the potential of citizen science to reduce labour costs depends on how projects are designed and are set up and for what purpose [87]. For example, while the co-design of citizen science may result in relatively high costs per observation [87], linking citizen science and citizen observatories with hydrological modelling to raise awareness of flood hazards and to facilitate two-way communication between citizens and local authorities has the potential not only to reduce flood risk but also to reduce damage by 45% compared to a business as usual scenario [88].

National (open) science policies present an opportunity to mitigate this misconception by explicitly framing the value of open societal engagement in science alongside robust support for professional science practices. Further, such framing will enhance the scientific value of citizen science engagement; in many fields, citizen science is demonstrated to have the greatest impact when it complements, rather than replaces, professional scientific activity (e.g. [87,88]).

Vohland *et al*. [86] and Mirowsky [23] have highlighted the risk of how the skills, time and commitment of citizen scientists can also be exploited to save costs. Additionally, box-ticking exercises on public participation in research projects can also be instrumentalized to help with compliance with the requirements of funders to achieve social impact targets and international agreements benchmarks. Policies can play a critical role in supporting the emergence of public participation while establishing standards and indicators to minimize the risks of exploitation.

For this reason, it is essential that the discussion on the need to focus on the research process, not just on outcomes, continues. Responsible Research and Innovation principles are important for guiding the development of evaluation indicators that avoid the instrumentalization of citizen engagement in science [89]. Stakeholders and funders must always consider who will benefit from open engagement and need to acknowledge and accommodate, where possible, that invited communities' own priorities are taken into account and receive reciprocal benefit from participation.

### 4.3. Diversity, equity and inclusion in open engagement

A parallel challenge that cannot be ignored is how the UNESCO Recommendation on Open Science can be implemented in an inclusive way, so that active citizen scientists reflect the diversity of society [24,90]. Currently, various biases are prevalent in citizen science participation, including age, gender, ethnicity and socioeconomic status [91]. Particular emphasis and funding must therefore be directed at increasing participation from groups of society that for lack of education, confidence, time or financial opportunities might otherwise not engage in scientific processes, even if open. This is particularly important in the increasing digitalization of society, as existing socio-economic challenges are in some cases aggravated by the digital divide. At a global level, access barriers that may limit scientists from economically disadvantaged countries from fully participating in open science, including limited technological and computational resources, disproportionate opportunity costs and lack of career benefit, must be recognized and addressed.

### 4.4. Impact and evaluation of open engagement of societal actors

Open societal engagement impact must be documented and disseminated to evidence its value and support the case for its adoption [92,93]. Relevant scientific data and actionable insights will reach their highest impact when they are open, accessible and easy to understand for all actors. Best practice and key case studies help highlight best practice within and across relevant agencies. For example, consolidation projects such as WeObserve enabled the analysis, documentation and dissemination of best practice of four European Commission-funded Citizen Observatory projects, encapsulated in the WeObserve Roadmap and Cookbook, which now serve to further the state of the art [40].

In terms of wider impacts, changing interactions between science and society have long been conceptualized and analysed from a variety of perspectives, none of which is uncontested [94]. They point to particular approaches for conducting, organizing and evaluating knowledge production that is focused on interactions between scientific disciplines, on societal relevance and on interactive relationships between science, industry, and the public sector (e.g. Mode 2 of knowledge production [95]; post-academic science [96]; Triple Helix [97,98]) and even with civil society (quadruple helix [99,100]). In promoting the open engagement of societal actors in science, the UNESCO Recommendation, embeds and at the same time stretches these conceptualizations further: with an integral role for citizens and communities in science as knowledge holders and co-producers; with potential changes in leadership (i.e. scientific activities are no longer necessarily led by researchers); and with interactions along any step of the scientific method, i.e. from the beginning of knowledge production (to identify new research problems, collect new data and create new knowledge), rather than just at the end. Open engagement of societal actors requires us to rethink the impacts of science on society and helps transform impact assessment of such joint knowledge production, moving from a linear model into a more connected and interwoven cycle of knowledge co-production with diverse actors.

Open science approaches—such as citizen science, the development of open-code software and free hardware projects, and incipient experiments in the open evaluation of research work—involve many invisible and less visible practices that cannot be as easily grasped through traditional metrics and quantitative indicators that focus on the evaluation of research results rather than the research process. There is a need to move to a wider set of evaluation indicators that focus on the research process, not just the outcomes at the end of it [6,92,93]. The current effort to identify alternative indicators for excellence and impacts has established that the impact of research must go beyond commercialization and industrial impact [101], and this broader understanding of impact must be integrated into all aspects of the research process [102], critically reflecting on whether alternative metrics (altmetrics data such as social media counts) reflect public interest and discussion of research rather than societal impact [103] and the extent to which they can foster open engagement of societal actors Research on the best process for generating indicators for the overall monitoring framework of the UNESCO Recommendation on Open Science may be best done in the spirit of open science, using a co-design approach with relevant stakeholder groups. A useful example of how agreed principles for governments to design and implement effective, efficient, and inclusive policies can be accompanied by indicator-based monitoring is constituted by the indicator framework [104] for the OECD Water Governance Principles [105].

Appropriate methods to measure the impact of societal engagement are emerging. H2020 projects in Europe such as Making Sense, GROW Observatory, Ground Truth 2.0 and MICS, have started to develop metrics and instruments to capture and evaluate citizen science impacts [49,51,92,93,106]. These metrics can be adapted to other open engagement policies and projects. Citizen science can facilitate new narratives on how open and participatory science as a policy tool can empower policymakers to generate more societal impact by engaging with citizens and science in a systematic way. Citizen science can also contribute to monitoring progress towards the SDGs beyond quantitative data contributions for indicator monitoring, as participatory approaches can help translate the SDG global framework and message to local realities [49,106].

All the aspects discussed in this opportunities and challenges section highlight the extent of the cultural transition and attitude change required from different stakeholder groups (funders, academics, policy makers, policy evaluators, etc). Apart from being multi-stakeholder, the change must also be multilevel, taking into account the entire policy process, from the policy formulation stage, to its evaluation. As a first step, adequate (open) science policies can set the conditions for this change to happen at the national level, and trigger the enabling conditions at the regional and local levels.

## 5. Conclusion

This work has identified the need for new ways of enabling and monitoring open engagement of societal actors in science. We have discussed what the results mean for monitoring and evaluating the open engagement of societal actors via national and regional (open) science policies. Thus, challenging and exciting work lies ahead. An essential next step concerns the practical process at national level that evolves existing science policy into proactive open science policies. Key insights and approaches for how to manage such processes effectively are emerging, including from the EU Mutual Learning Exercise. The recommendations from the latter offer a complementary tool to the building blocks presented in this paper, jointly creating a pathway for an enabling environment for the open engagement of societal actors. With the lead of UNESCO, the development of theoretical and methodological contributions will support countries to expand their science policy to include and champion not only open data but open engagement principles.

## Data Availability

Wehn U *et al.* 2024 Collected insights and recommendations for shaping (open) science policies to ensure open engagement of societal actors in science [Dataset]. Dryad. https://doi.org/10.5061/dryad.rbnzs7hkp [107].

## References

[1] Nielsen M. 2011 Reinventing Discovery: The New Era of Networked Science. In Reinventing discovery. Princeton University Press.

[2] Boulton G, Campbell P, Collins B, Elias P, Hall W, Laurie G, Walport M. 2012 The Royal Society.

[3] Burgelman JC, Pascu C, Szkuta K, Von Schomberg R, Karalopoulos A, Repanas K, Schouppe M. 2019 Open science, open data, and open scholarship: European policies to make science fit for the twenty-first century. Frontiers in Big Data **2**, 43. (10.3389/fdata.2019.00043) 33693366 PMC7931888

[4] European Commission, Directorate-General for Research and Innovation. Mendez E, Lawrence R. 2020 Progress on open science : towards a shared research knowledge system : final report of the open science policy platform, (R. Lawrence, editor). Luxembourg: Publications Office. See https://data.europa.eu/doi/10.2777/00139.

[5] UNESCO. n.d. Recommendations. (accessed 30 Mar 2023). See https://www.unesco.org/en/legal-affairs/standard-setting/recommendations.

[6] De Filippo D, Sastrón-Toledo P. 2023 Influence of research on Open Science in the public policy sphere. Scientometrics **128**, 1995-2017.

[7] Ministry of Education and Culture. 2014 Open Science and Research Initiative 2014–2017. The Open Science and Research Roadmap. Reports of the Ministry of Education and Culture, Finland, 2014:21. Retrieved from http://julkaisut.valtioneuvosto.fi/bitstream/handle/10024/75210/okm21.pdf.

[8] Forsström PL, Haataja J. 2016 Open Science as an Instrument for Effective Research. Signum.

[9] Van Wezenbeek WJSM, Touwen HJJ, Versteeg AMC, van Wesenbeeck A. 2017 Nationaal plan open science. 10.4233/uuid:9e9fa82e-06c1-4d0d-9e20-5620259a6c65.

[10] MCTES. 2016 Ciência Aberta, Conhecimento para todos: Princípios orientadores. Ministério da Ciência, Tecnologia e Ensino Superior. Retrieved from Foster Blog. https://www.fosteropenscience.eu/content/ciencia-aberta-conhecimento-para-todos.

[11] Ministère de l'enseignement supérieur de la recherche. 2021 Second French Plan for Open Science. Generalizing open science in France 2021-2024 (accessed 30 Mar 2023). https://www.ouvrirlascience.fr/wp-content/uploads/2021/10/Second_French_Plan-for-Open-Science_web.pdf.

[12] Athanasiou S et al. 2020 National Plan for Open Science. Zenodo (accessed 30 Mar 2023)

[13] Rossi G, Caso R, Castelli D, Giglia E. 2021 National Research Programme 2021–2027. Italian National Plan for Open Science (accessed 8 Feb 2024). https://www.mur.gov.it/sites/default/files/2023-01/PNSA_2021-27_ENG.pdf.

[14] Bundesminiterium Bildung, Wissenschaft und Forshung. 2022 Open Science Policy Austria (accessed 8 Feb 2024). https://www.bmbwf.gv.at/Themen/HS-Uni/Hochschulgovernance/Leitthemen/Digitalisierung/Open-Science/Open-Science-Policy-Austria.html.

[15] MICINN. 2023 National Strategy for Open Science 2023–2027. Ministerio de Ciencia e Innovación. https://www.ciencia.gob.es/InfoGeneralPortal/documento/e5b759a4-d756-4af9-89b0-a8cf5fd28e20 (accessed 15 May 2024).

[16] MINECO. 2017 Plan Estatal de Investigación Científica y Técnica y de Innovación 2017–2020 (PEICTI). Ministerio de Economía, Industria y Competitividad (accessed 14 Mar 2023). https://www.aei.gob.es/sites/default/files/page/field_file/2021-10/PlanEstatalIDI.pdf.

[17] Boletín Oficial del Estado. 2022 Ley 17/2022, de 5 de septiembre, por la que se modifica la Ley 14/2011, de 1 de junio, de la Ciencia, la Tecnología y la Innovación (accessed 30 Mar 2023). https://www.boe.es/eli/es/l/2022/09/05/17/con.

[18] Mačiulienė M. 2022 Beyond open access: conceptualizing Open Science for knowledge co-creation. Frontiers in Communication **7**, 1-8. (10.3389/fcomm.2022.907745)

[19] Golumbic YN, Orr D, Baram-Tsabari A, Fishbain B. 2017 Between vision and reality: A study of scientists' views on citizen science. Citizen Science: Theory and Practice **2**, 6. (10.5334/cstp.53)

[20] Riesch H, Potter C. 2014 Citizen science as seen by scientists: Methodological, epistemological and ethical dimensions. Public Underst. Sci. **23**, 107-120. (10.1177/0963662513497324) 23982281

[21] Ross-Hellauer T. 2022 Open science, done wrong, will compound inequities. Nature **603**, 363. (10.1038/d41586-022-00724-0) 35288691

[22] Mirowski P. 2018 The future (s) of Open Science. Soc. Stud. Sci. **48**, 171-203. (10.1177/0306312718772086) 29726809

[23] Mirowski P. 2017 Against citizen science (accessed 13 Mar 2023). https://aeon.co/essays/is-grassroots-citizen-science-a-front-for-big-business.

[24] Cooper CB et al. 2021 Inclusion in citizen science: The conundrum of rebranding. Science **372**, 1386-1388. (10.1126/science.abi6487)

[25] Haklay M et al. 2021 Contours of citizen science: a vignette study. R. Soc. Open Sci. **8**, 202108. (10.1098/rsos.202108) 34457323 PMC8385385

[26] Wehn U, Gobel C, Bowser A, Hepburn L, Haklay M. 2020 Global citizen science perspectives on open science. CSGP Citizen Science & Open Science Community of Practice. Short Paper for UNESCO Advisory Body on the Open Science Recommendation, May. https://osf.io/6qjyg/.

[27] Haklay M et al. 2020 ECSA's Characteristics of Citizen Science. Version 1. Berlin, Germany, European Citizen Science Association (ECSA), 6pp. (10.5281/zenodo.3758668)

[28] Strasser B, Baudry J, Mahr D, Sanchez G, Tancoigne E. 2019 ‘Citizen Science’? Rethinking Science and Public Participation. Science & Technology Studies **32**, 52-76. (10.23987/sts.60425)

[29] Israel B, Schulz A, Parker E, Becker A. 2001 Community-based participatory research: policy recommendations for promoting a partnership approach in health research. Educ. Health **14**, 182-197. (10.1080/13576280110051055) 14742017

[30] Wandersman A. 2003 Community science – Bridging the Gap between Science and Practice with Community-centred Models. Am. J. Community Psychol. **31**, 227-242. (10.1023/A:1023954503247) 12866681

[31] Danielsen F, Eicken H, Funder M, Johnson N, Lee O, Theilade I, Argyriou D, Burgess ND. 2022 Community Monitoring of Natural Resource Systems and the Environment. Annual Review of Environment and Resources **47**, 637-670. (10.1146/annurev-environ-012220-022325)

[32] Hager G et al. 2021 Onto new horizons: insights from the WeObserve project to strengthen the awareness, acceptability and sustainability of Citizen Observatories in Europe. JCOM: Journal of Science Communication **20**, A01.

[33] Ottinger G. 2017 Crowdsourcing Undone Science, Engaging Science. Technology and Society **3**, 560-574. (10.17351/ests2017.124)

[34] Guo B, Chen C, Zhang D, Yu Z, Chin A. 2016 Mobile crowd sensing and computing: when participatory sensing meets participatory social media. IEEE Commun. Mag. **54**, 131-137. (10.1109/MCOM.2016.7402272)

[35] Argyris C, Schön SA. 1989 Participatory Action Research and Action Science Compared: A Commentary. American Behavioral Scientist **32**, 612-623. (10.1177/0002764289032005008)

[36] Leydesdorff L, Ward J. 2005 Science shops: a kaleidoscope of science–society collaborations in Europe. Public Underst. Sci. **14**, 353-372. (10.1177/0963662505056612)

[37] Ståhlbröst A. 2012 A set of key principles to assess the impact of Living Labs. International Journal of Product Development **17**, 60-75. (10.1504/IJPD.2012.051154)

[38] Wehn U, Hsing PY, Ajates R, Kragh G, Mandeville G. 2022 *What do we mean by the open engagement of societal actors & dialogues with other knowledge systems?* §2 in Wehn, U., & Hepburn, L. (2022). Guidance for the implementation of the UNESCO Open Science Recommendation re. ‘Opening science to society’ (FINAL), unpublished report, Zenodo. (10.5281/zenodo.7472827)

[39] Wehn U. 2022 Citizen Science for co-monitoring and co-managing impact on ecosystems and inland waters, chapter in. In Encyclopedia of inland waters, second edition, §5 - human pressures and management of inland waters (eds K Tockner, T Mehner), pp. 35-36. Elsevier. (10.1016/b978-0-12-819166-8.00184-5)

[40] Hager G et al. 2021 Roadmap for the uptake of the Citizen Observatories' knowledge base. In: WeObserve policy webinar organised by the European Commission, DG Research & Innovation, Directorate Healthy Planet, B3 – Sector Environmental Observation, 18 June 2021.

[41] Wehn U, Hepburn L. 2022 Guidance for the implementation of the UNESCO Open Science Recommendation re. ‘Opening science to society’ (FINAL), unpublished report. Zenodo. (10.5281/zenodo.7472827)

[42] UNESCO. 2021 UNESCO Recommendation on Open Science (accessed 28 Mar 2023). https://unesdoc.unesco.org/ark:/48223/pf0000379949.locale=en.

[43] Wehn U, Ajates R, Kragh G, Mandeville G, Somerwill L, Kiefer S. 2022 *Recommendations for opening science to society via Open Science policy and for monitoring of policy implementation*, §3 in Wehn, U., & Hepburn, L. (2022). Guidance for the implementation of the UNESCO Open Science Recommendation re. ‘Opening science to society’ (FINAL), unpublished report, Zenodo. (10.5281/zenodo.7472827) PMC11285865 39076808

[44] McKinley DC et al. 2017 Citizen science can improve conservation science, natural resource management, and environmental protection. Biological Conservation **208**, 15-28. (10.1016/j.biocon.2016.05.015)

[45] Notermans VI, Montanari MC, Janssen A, Hölscher K, Wittmayer JM, Passani A. 2022 Recommendations to mainstream citizen science in policy. ACTION project. (10.5281/zenodo.5772236)

[46] Pandya RE. 2012 A framework for engaging diverse communities in citizen science in the US. Frontiers in Ecology and the Environment **10**, 314-317. (10.1890/120007)

[47] Chan L, Hall B, Piron F, Tandon R, Williams L. 2020 Open science beyond open access: For and with communities. A step towards the decolonization of knowledge. *Ottawa: The Canadian commission for UNESCO's IdeaLab*.

[48] Danielsen F, Burgess ND, Jensen PM, Pirhofer-Walzl K. 2010 Environmental monitoring: the scale and speed of implementation varies according to the degree of peoples involvement. Journal of Applied Ecology **47**, 1166-1168. (10.1111/j.1365-2664.2010.01874.x)

[49] Ajates R, Hager G, Georgiadis P, Coulson S, Woods M, Hemment D. 2020 Local action with global impact: The case of the GROW observatory and the sustainable development goals. Sustainability **12**, 10518. (10.3390/su122410518)

[50] NEJAC. 2017 Recommendations and Guidance for EPA to Develop Monitoring Programs in Communities (accessed 30 Mar 2023). https://www.epa.gov/sites/default/files/2018-01/documents/monitoring-final-10-6-17.pdf.

[51] Warin C, Delaney N. 2020 Citizen science and citizen engagement. Achievements in Horizon 2020 and recommendations on the way forward. Directorate-General for Research and Innovation Science with and for society.

[52] Shanley LA, Michelucci P, Tsosie K, Wyeth G, Drapkin JK, Azelton K, Cavalier D, Holmberg J. 2021 Public Comment on Draft NOAA citizen science Strategy. Human Computation **8**, 25-42. (10.15346/hc.v8i1.130)

[53] Coulson S, Woods M, Making Sense EU. 2021 Citizen Sensing: An action-orientated framework for citizen science. Frontiers in Communication **6**, 629700. (10.3389/fcomm.2021.629700)

[54] Brondízio ES et al. 2021 Locally based, regionally manifested, and globally relevant: Indigenous and local knowledge, values, and practices for nature. Annual Review of Environment and Resources **46**, 481-509. (10.1146/annurev-environ-012220-012127)

[55] Reyes-García V, Tofighi-Niaki A, Austin BJ, Benyei P, Danielsen F, Fernández-Llamazares Á, Sharma A, Soleymani-Fard R, Tengö M. 2022 Data sovereignty in community-based environmental monitoring: toward equitable environmental data governance. BioScience **72**, 714-717. (10.1093/biosci/biac048) 35923191 PMC9343228

[56] Carroll SR et al. 2020 The CARE principles for indigenous data governance. Data Science Journal. 19. 43pp. (doi:10.5334/dsj-2020-043)

[57] Bonn A et al. 2022 White paper citizen science strategy 2030 for Germany. Helmholtz Association, Leibniz Association, Fraunhofer Society, Universities and Non-Academic Institutions. (10.5281/zenodo.7117771)

[58] de la Torre EM, Sandoval Hamón LA, Galindo R, Casani F. 2021 Análisis los estándares, regulaciones, políticas y estrategias (tanto nacionales como internacionales) sobre ciencia abierta en la educación superior–entregable 1. Zenodo. (10.5281/zenodo.4882885)

[59] EPA. 2018 Information to Action Strengthening EPA citizen science Partnerships for Environmental Protection (accessed 3 Feb 2022). https://www.epa.gov/sites/default/files/2020-04/documents/nacept_2018_citizen_science_publication_eng_final_v3_508.pdf.

[60] Johnson N, Druckenmiller ML, Danielsen F, Pulsifer PL. 2021 The use of digital platforms for community-based monitoring. BioScience **71**, 452-466. (10.1093/biosci/biaa162) 33986630 PMC8106997

[61] Dominik M, Nzweundji JG, Ahmed N, Carnicelli S, Mat Jalaluddin NS, Rivas DF, Narita V, Enany S, Rojas CR. 2022 Open Science–for whom? Data Science Journal **21**, 1.

[62] European Commission, Directorate-General for Research and Innovation. 2021 Enabling open science and societal engagement in research, publications office. https://data.europa.eu/doi/10.2777/057047.

[63] European Commission, Directorate-General for Research and Innovation, Haklay M. 2022 Open science and intellectual property rights : How can they better interact? : state of the art and reflections : executive summary, Publications Office of the European Union, 2022. See https://data.europa.eu/doi/10.2777/347305

[64] Tyson E, Rejeski D. 2016 Strategic Recommendations for Federal citizen science and Crowdsourcing (accessed 13 Mar 2023). https://www.wilsoncenter.org/article/strategic-recommendations-for-federal-citizen-science-and-crowdsourcing.

[65] Barkved LJ, Furuseth IS, Langaas S. 2020 Mulig bruk av folkeforskning og nettdugnad i vannforvaltningen. NIVA-rapport.

[66] Museum für Naturkunde. 2020 Our world – our goals: citizen science for the Sustainable Development Goals (accessed 3 Feb 2023). https://www.museumfuernaturkunde.berlin/en/press/press-releases/our-world-our-goals-citizen-science-sustainable-development-goals.

[67] García-Holgado A, García-Peñalvo FJ, Butler P. 2020 Technological ecosystems in citizen science: A framework to involve children and young people. Sustainability **12**, 1863. (10.3390/su12051863)

[68] Miczajka VL, Klein AM, Pufal G. 2015 Elementary school children contribute to environmental research as citizen scientists. PLoS ONE **10**, e0143229. (10.1371/journal.pone.0143229) 26581087 PMC4651542

[69] Roche J et al. 2020 Citizen science, education, and learning: challenges and opportunities. Frontiers in Sociology **5**, 613814. (10.3389/fsoc.2020.613814) 33869532 PMC8022735

[70] Woods M, Hemment D, Ajates-Gonzalez R. 2019 A Participant-Centred Model for Citizen Observatories at Scale. In Geophysical Research Abstracts (Vol. 21).

[71] European Commission, Directorate-General for Research and Innovation, Gold M, Arias R, Haklay M, Irwin A, Mazzonetto M, Meijer I, Radicchi A, Leo G, Arentoft M. 2023 Mutual learning exercise – Citizen science initiatives – Policy and practice – Final report, Publications Office of the European Union. See https://data.europa.eu/doi/10.2777/988919

[72] European Commission, Directorate-General for Research and Innovation, Chan, T. 2019 Open research policies in the United Kingdom, Publications Office. See https://data.europa.eu/doi/10.2777/24416.

[73] Saunders TE. 2022 The Future is Open Establishing Wider Open Access for Research Publications in Aotearoa New Zealand. Office of the Prime Minister's Chief Science Advisor. Intern Report. Non-peer-reviewed - Version 1–31 May 2022 (accessed 28 Mar 2023). See https://www.pmcsa.ac.nz/2022/07/15/the-future-is-open-intern-report-on-open-access-publishing-in-aotearoa/.

[74] Sánchez F, De Filippo D. 2022 Informe Sobre Los Conocimientos, Actitudes Y Valoraciones De La Ciencia Abierta. Análisis De Los Procedimientos, Barreras, Limitaciones, Elementos Facilitadores Para Fomentar La Ciencia Abierta En Las Universidades. Entregable 4. Zenodo. (10.5281/zenodo.6509944)

[75] CONACYT. 2018 Libro Blanco: Política Pública Ciencia Abierta (accessed 30 Mar 2023). https://conacyt.mx/wp-content/uploads/transparencia/planes_programas_informes/libros_blancos/Ciencia_Abierta.pdf.

[76] WeObserve consortium. 2021 WeObserve Cookbook: Guidelines for creating successful and sustainable Citizens Observatories. See www.weobserve.eu/weobserve-cookbook.

[77] Heberling JM, Miller JT, Noesgaard D, Weingart SB, Schigel D. 2021 Data integration enables global biodiversity synthesis. Proc. Natl Acad. Sci. USA **118**, e2018093118. (10.1073/pnas.2018093118) 33526679 PMC8017944

[78] Lazzeri E et al. 2021 ICDI Competence Centre for Open Science, FAIR and EOSC - Mission, Strategy and Action Plan. Zenodo. (10.5281/zenodo.5512638)

[79] ITK, Aarhus City Lab. 2022 Aarhus Citizen Science Guide. Aarhus Municipality (accessed 30 Mar 2023). https://aarhuscitylab.dk/media/83420/aarhus-citizen-science-guide-a5-webt.pdf.

[80] MICINN. 2020 EECTI. Estrategia Española de Ciencia, Tecnología e Innovación: 2021–2027. Ministerio de Ciencia e Innovación.

[81] Gold M, Wehn U. 2020 Mission Sustainable: Fostering an enabling environment for sustainable Citizen Observatories. In WeObserve policy brief 2. European citizen science Association Conference 2020: Encounters in citizen science.

[82] Haklay ME. 2015 Citizen science and policy: A European perspective. Washington, DC: Woodrow Wilson International Center for Scholars, 2015.

[83] Leonelli S. 2017 Mutual learning exercise: Open science—altmetrics and rewards incentives and rewards to engage in open science activities. no. Thematic Report, (3). European Commission Directorate-General for Research and Innovation. https://projects.research-and-innovation.ec.europa.eu/sites/default/files/rio/report/MLE-OS-Report-3%2520.pdf.

[84] Tancoigne E. 2019 Invisible brokers:’ citizen science’ on Twitter. JCOM-Journal of Science Communication **18**, A05.

[85] Latham A, Layton J. 2019 Social infrastructure and the public life of cities: Studying urban sociality and public spaces. Geography Compass **13**, e12444. (10.1111/gec3.12444)

[86] Vohland K, Weißpflug M, Pettibone L. 2019 Citizen science and the neoliberal transformation of science–An ambivalent relationship. Citizen Science: Theory and Practice 4(1): 25, pp. 1–9. (10.5334/cstp.186)

[87] Alfonso L, Gharesifard M, Wehn U. 2022 Analysing the value of environmental citizen-generated data: Complementarity and cost per observation. J. Environ. Manage. **303**, 114157. (10.1016/j.jenvman.2021.114157) 34839172

[88] Ferri M, Wehn U, See L, Monego M, Fritz S. 2020 The value of citizen science for flood risk reduction: Cost-benefit analysis of a citizen observatory in the Brenta-Bacchiglione catchment. Hydrology and Earth System Sciences **24**, 5781-5798. (10.5194/hess-24-5781-2020)

[89] Smallman M. 2018 Citizen science and responsible research and innovation. In Citizen Science: Innovation in Open Science, Society and Policy (eds S Hecker, M Haklay, A Bowser, Z. Makuch, J Vogel, A Bonn), pp. 241–253. London: UCL Press. http://www.jstor.org/stable/j.ctv550cf2.24.

[90] Blake C, Rhanor A, Pajic C. 2020 The demographics of citizen science participation and its implications for data quality and environmental justice. Citizen Science: Theory and Practice **5**, 21. (10.5334/cstp.320)

[91] Pateman RM, Dyke A, West SE. 2021 The diversity of participants in environmental citizen science. Citizen Science: Theory and Practice. **6**, 9. (10.5334/cstp.369)

[92] Wehn U et al. 2021 Capturing and communicating impact of citizen science for policy: A storytelling approach. J. Environ. Manage. **295**, 113082. (10.1016/j.jenvman.2021.113082) 34167062

[93] Wehn U et al. 2021 Impact assessment of citizen science: state of the art and guiding principles for a consolidated approach. Sustainability Sci. **16**, 1683-1699. (10.1007/s11625-021-00959-2)

[94] Hessels LK, Van Lente H. 2008 Re-thinking new knowledge production: A literature review and a research agenda. Evol. Hum. Behav. **37**, 740-760. (10.1016/j.respol.2008.01.008)

[95] Gibbons M, Limoges C, Nowotny H, Schwartzman S, Scott P, Trow M. 1994 The New production of knowledge: The dynamics of science and research in contemporary societies. London: SAGE.

[96] Ziman J. 2000 Real science: what it is, and what it means. Cambridge: Cambridge University Press.

[97] Etzkowitz H, Leydesdorff L. 2000 The dynamics of innovation: from National Systems and ‘Mode 2’ to a Triple Helix of university–industry–government relations. Evol. Hum. Behav. **29**, 109-123. (10.1016/S0048-7333(99)00055-4)

[98] Leydesdorff L, Meyer M. 2006 Triple Helix indicators of knowledge-based innovation systems: introduction to the special issue. Evol. Hum. Behav. **35**, 1441-1449. (10.1016/j.respol.2006.09.016)

[99] Carayannis EG, Rakhmatullin R. 2014 The quadruple/quintuple innovation helixes and smart specialisation strategies for sustainable and inclusive growth in Europe and Beyond. Journal of the Knowledge Economy **5**, 212-239. (10.1007/s13132-014-0185-8)

[100] Ivanova I. 2014 Quadruple helix systems and symmetry: a step towards helix innovation system Classification. Journal of the Knowledge Economy **5**, 357-369. (10.1007/s13132-014-0201-z)

[101] Perkmann M, Salandra R, Tartari V, McKelvey M, Hughes A. 2021 Academic engagement: A review of the literature 2011–2019. Evol. Hum. Behav. **50**, 104114. (10.1016/j.respol.2020.104114)

[102] Owen R. 2021 Enabling open science and societal engagement in research. Luxemburg: Publications Office of the European Union. https://data.europa.eu/doi/10.2777/057047.

[103] Tahamtan I, Bornmann L. 2020 Altmetrics and societal impact measurements: Match or mismatch? A literature review. El profesional de la información **29**, e290102. (10.3145/epi.2020.ene.02)

[104] OECD. 2018 Implementing the OECD principles on water governance: indicator framework and evolving practices, OECD studies on water. Paris: OECD Publishing. (10.1787/9789264292659-en)

[105] OECD. 2015 OECD principles on water governance. Paris: OECD. See www.oecd.org/gov/regional-policy/OECD-Principles-on-Water-Governancebrochure.pdf.

[106] Sprinks J, Woods SM, Parkinson S, Wehn U, Joyce H, Ceccaroni L, Gharesifard M. 2021 Coordinator perceptions when assessing the impact of citizen science towards sustainable development goals. Sustainability **13**, 2377. (10.3390/su13042377)

[107] Wehn U et al. 2024 Collected insights and recommendations for shaping (open) science policies to ensure open engagement of societal actors in science [Dataset]. Dryad. (10.5061/dryad.rbnzs7hkp)

